# Brain correlates of declarative memory atypicalities in autism: a systematic review of functional neuroimaging findings

**DOI:** 10.1186/s13229-022-00525-2

**Published:** 2023-01-10

**Authors:** Pierre Desaunay, Bérengère Guillery, Edgar Moussaoui, Francis Eustache, Dermot M. Bowler, Fabian Guénolé

**Affiliations:** 1 Service de Psychiatrie de l’Enfant et de l’Adolescent, CHU de Caen Normandie, 27 rue des compagnons, 14000 Caen, France; 2 EPHE, INSERM, U1077, Pôle des Formations et de Recherche en Santé, CHU de Caen Normandie, GIP Cyceron, Neuropsychologie et Imagerie de la Mémoire Humaine, Normandie Univ, UNICAEN, PSL Research University, 2 rue des Rochambelles, 14032 Caen Cedex CS, France; 3 Autism Research Group, City University of London, DG04 Rhind Building, Northampton Square, EC1V 0HB London, UK; 4 Faculté de Médecine, Pôle des Formation et de Recherche en Santé, Université de Caen Normandie, 2 rue des Rochambelles, 14032 Caen cedex CS, France

**Keywords:** Autism spectrum disorder, Brain, Electroencephalography, Electrophysiology, Magnetic resonance imaging, Magnetoencephalography, Memory, Neuroimaging

## Abstract

The long-described atypicalities of memory functioning experienced by people with autism have major implications for daily living, academic learning, as well as cognitive remediation. Though behavioral studies have identified a robust profile of memory strengths and weaknesses in autism spectrum disorder (ASD), few works have attempted to establish a synthesis concerning their neural bases. In this systematic review of functional neuroimaging studies, we highlight functional brain asymmetries in three anatomical planes during memory processing between individuals with ASD and typical development. These asymmetries consist of greater activity of the left hemisphere than the right in ASD participants, of posterior brain regions—including hippocampus—rather than anterior ones, and presumably of the ventral (occipito-temporal) streams rather than the dorsal (occipito-parietal) ones. These functional alterations may be linked to atypical memory processes in ASD, including the pre-eminence of verbal over spatial information, impaired active maintenance in working memory, and preserved relational memory despite poor context processing in episodic memory.

## Highlights

- Brain correlates of memory processes in autism spectrum disorder display functional asymmetries.
- Greater reliance on left than right-hemisphere functioning.
- Greater reliance on posterior than anterior brain functioning.
- Greater reliance on ventral stream than dorsal stream functioning.
- These functional asymmetries may account for memory atypicalities in ASD.

## Introduction

Autism spectrum disorder (ASD) is a lifelong neurodevelopmental disorder comprising difficulties in social communication and interaction, restricted or repetitive behaviors or interests, alongside sensory characteristics. Autism-specific characteristics in the cognitive and sensory domains are thought to result in atypical memory development in this population, which has been noted since the first clinical descriptions of the disorder [1–3]. In addition, memory abilities help supporting cognitive rehabilitation in ASD [4]. Understanding memory is therefore of significant interest in ASD, and in this sense, several cognitive models have been developed over time [5]. This contrasts with the lack of any systematic neural model, despite a growing number of publications in this area.

Memory encompasses a complex set of cognitive functions and multicomponent systems. Short-term memory stores a limited quantity of information [6], and this has been further extended by Baddeley’s model of working memory (WM) [7], which emphasizes the manipulation of information during cognitive tasks and encompasses two modality-specific short-term stores (visuospatial sketchpad and phonological loop) that depend on a central executive. WM involves several cognitive processes: (1) selective attention to perceptual information and activation of semantic representations, during encoding; (2) sustained attention, rehearsal, and inhibition of task-irrelevant information, during maintenance; and (3) selective attention and pattern completion, during retrieval [8]. By contrast, long-term memory (LTM) contains unlimited quantities of information held for long durations and includes two subsystems, namely the semantic memory that contains general knowledge and is associated with noetic awareness and the episodic memory. Most studies conducted in ASD have focused on episodic LTM, which consists of specific memories of personally experienced events, situated in the temporal and spatial contexts of their acquisition. Episodic LTM is associated with autonoetic awareness and recollection. Episodic LTM is associated with autonoetic awareness and recollection. During encoding and retrieval, episodic LTM involves similar cognitive processes to those seen in WM, with the difference between the two types of memory being found mainly in the way information is stored [9, 10]. In WM, storage is kept active by means of rehearsal, while storage is supported by the semantic memory system in episodic LTM [11].

Neuroimaging studies in memory research either can evaluate brain activity during memory tasks compared with control conditions, or can explore functional connectivity, i.e., the temporal correlations between spatially remote neurophysiological events. The imaging methods used are functional magnetic resonance imaging (fMRI) and electro-/magnetoencephalography (EEG/MEG): fMRI measures the BOLD (blood-oxygen-level-dependent) signal of cortical and subcortical regions with high spatial precision and EEG/MEG measures electrical activity near the cortical surface from pyramidal neurons firing simultaneously with high temporal and spectral resolutions [12].

For WM, neuroimaging studies in typical development (TD) have evidenced a persistent activation and functional connectivity in the absence of stimuli, between fronto-parietal and posterior areas [8]. Activation of the prefrontal cortex and particularly the dorsolateral part have been associated with rehearsal and manipulation of information, and activity in parietal areas with attentional processes [13]. Activation in posterior areas has been associated with WM storage, especially within the same specialized perceptual areas that are recruited during the processing of low-level features of items at encoding, e.g., early visual cortex for visual information [14], left parietal areas for verbal ones [15], which was theorized as the *sensory-recruitment hypothesis* [16, 17]. Moreover, activation in the fronto-parietal network is coupled with deactivation of the default-mode network (DMN) that comprises bilateral cortical areas activated during resting state and deactivated during any task, located in medial and lateral parietal, medial prefrontal, and medial and lateral temporal cortices [18]. Electrophysiological studies provide evidence that high-frequency gamma oscillations code for items, while long-range low-frequency theta oscillations are associated with the temporal organization of WM items during maintenance [19, 20]. Gamma cycles are nested within theta cycles during short-term maintenance (*nested cycles model*) [21], with the ratio of theta to gamma cycle length being predictive of WM capacity [22]. Low-frequency alpha oscillations have been associated with local (occipital) inhibition of task-irrelevant information [20] and also in WM maintenance at long-range scale [23].

Episodic LTM, by contrast, relies on the storage of information associated in TD with the medial temporal lobes and other widely distributed cortical areas depending on memory tasks. Based on fMRI data, the binding item-context account [24] posits that item and context representations are, respectively, supported by the perirhinal and parahippocampal cortices and associated in memory by the hippocampus, while the prefrontal cortex mainly dorsolateral part enables information processing during encoding and retrieval. Linking cognitive and neuroanatomical models, the *predictive interactive multiple memory systems* framework [25] emphasizes the functional connectivity between the hippocampus (associated with episodic memory), the perirhinal cortex (associated with semantic memory), and sensory cortices (associated with perceptual representation systems), during memory stages. Electrophysiological studies of episodic LTM mainly focus on recognition, allowing an analysis of memory processes. Event-related potential (ERP) studies describe old/new effects (consisting in a greater positivity for correctly recognized old items over correctly rejected new items) occurring on: early potentials (0–300 ms) related to priming, the FN400 potential (frontal, negative, around 300–500 ms) indexing familiarity, the LPC (late positive—or parietal—component, around 500–700 ms) indexing recollection, then late frontal negativity (indexing post-retrieval monitoring) [26–28]. These familiarity-related FN400 and recollection-related LPC potentials have been, respectively, associated with the semantic and episodic memory systems in the *dual-process theory of recognition* [29].

Behavioral studies in ASD primarily focus on episodic LTM, providing neuroanatomical hypotheses that have been assessed only to a limited extent. Greater difficulties have been reported for complex information processing, particularly in the visual modality [30, 31], suggesting reduced connectivity between frontal associative and posterior sensory areas [32]. Individuals with ASD generally draw less benefit from the categorical-semantic aspects of the to-be-remembered information [33, 34], suggesting diminished detection of higher-order similarities between related items of information, which possibly implicate frontal and medial temporal lobes [35]. It contrasts with typical levels of memory performance in situations that provide support for the processing of relational information (the *task support hypothesis*) [33, 36, 37]. Preserved item-specific and context-independent memory, alongside with greater difficulties in the recollection over familiarity process [38–40], suggested dysfunction of the hippocampus that supports binding [37, 41]. It has been more recently challenged by findings of a greater visual memory both for items and associations in ASD when supported by recollection than familiarity [42].

Recently, several meta-analyses have been conducted, highlighting a specific pattern in declarative memory in ASD. Two specific meta-analyses have been realized on WM [43, 44], one included WM and episodic LTM domains [5], and the two most recent focused on episodic LTM [45] and face recognition [46]. Meta-analyses on WM have, respectively, identified an overall medium effect size with greater difficulties for visuospatial WM compared with verbal WM [44], and medium to large effect sizes for both verbal WM and visuospatial WM [43]. One meta-analysis reported an overall medium effect size for WM, homogeneous whatever the type of material either verbal, visual, or spatial (with, however, a tendency for medium to large for the latter), along with overall low to medium effect size for episodic LTM, with a small effect size only for verbal LTM, a medium one for visual LTM (inconclusive results for visuospatial LTM), and a similar decrement for associative and non-associative memory [5]. Other meta-analyses on episodic LTM have, respectively, reported an overall low to medium effect size, with greater difficulties in ASD for complex stimuli (sentences and stories), compared with simple stimuli (words and pictures) [45], and large deficits for both face identity recognition and discrimination [46]. Hence, these results point toward greater difficulties for visuospatial material over verbal material, which are even larger for faces, and greater still for WM over episodic LTM.

In contrast to consistent cognitive models and behavioral meta-analyses of memory in ASD, reviews and syntheses are lacking for neuroimaging studies. Only two preliminary reviews have been conducted, concluding that there was reduced functional connectivity in WM in adolescents with ASD [47], and prefrontal cortex dysfunction as a general factor for episodic LTM difficulties in ASD [48]. To address this gap, we conducted a review of neuroimaging studies on memory in ASD, including both fMRI and electrophysiological studies. We focused on WM and episodic LTM studies that represent the core of studies on memory in ASD, distinguishing verbal material, and visuospatial material, including faces.

## Methods

This systematic review has adopted the Preferred Reporting Items for Systematic Reviews and Meta-Analyses guidelines [49]. The protocol of this review was prospectively registered in the *International Prospective Register of Systematic Reviews* (PROSPERO; CRD42020203766).

### Selection criteria

We selected studies with the following inclusion criteria: (1) studies comparing individuals with autism spectrum disorder (ASD) and those with TD, regardless of age, published in English and in peer-reviewed journals; (2) participants with autism presenting one of the spectrum phenotypes (i.e., autism, autism spectrum disorder, autistic disorder, Asperger syndrome, pervasive developmental disorders not otherwise specified); and (3) neuroimaging studies focusing on working- or episodic memory, using electrophysiological—EEG or MEG—or fMRI methods, with activation or connectivity measures.

Exclusion criteria were as follows: (1) animal research studies; (2) participants presenting autism associated with a known medical or genetic condition; (3) studied memories not being presented during the task, such as autobiographical memories; (4) studies on learning; (5) non-declarative memory tasks; and 6) neuroimaging not being performed during the memory task (brain/cognition covariance analyses).

### Information sources and search strategies

**Fig. 1:**
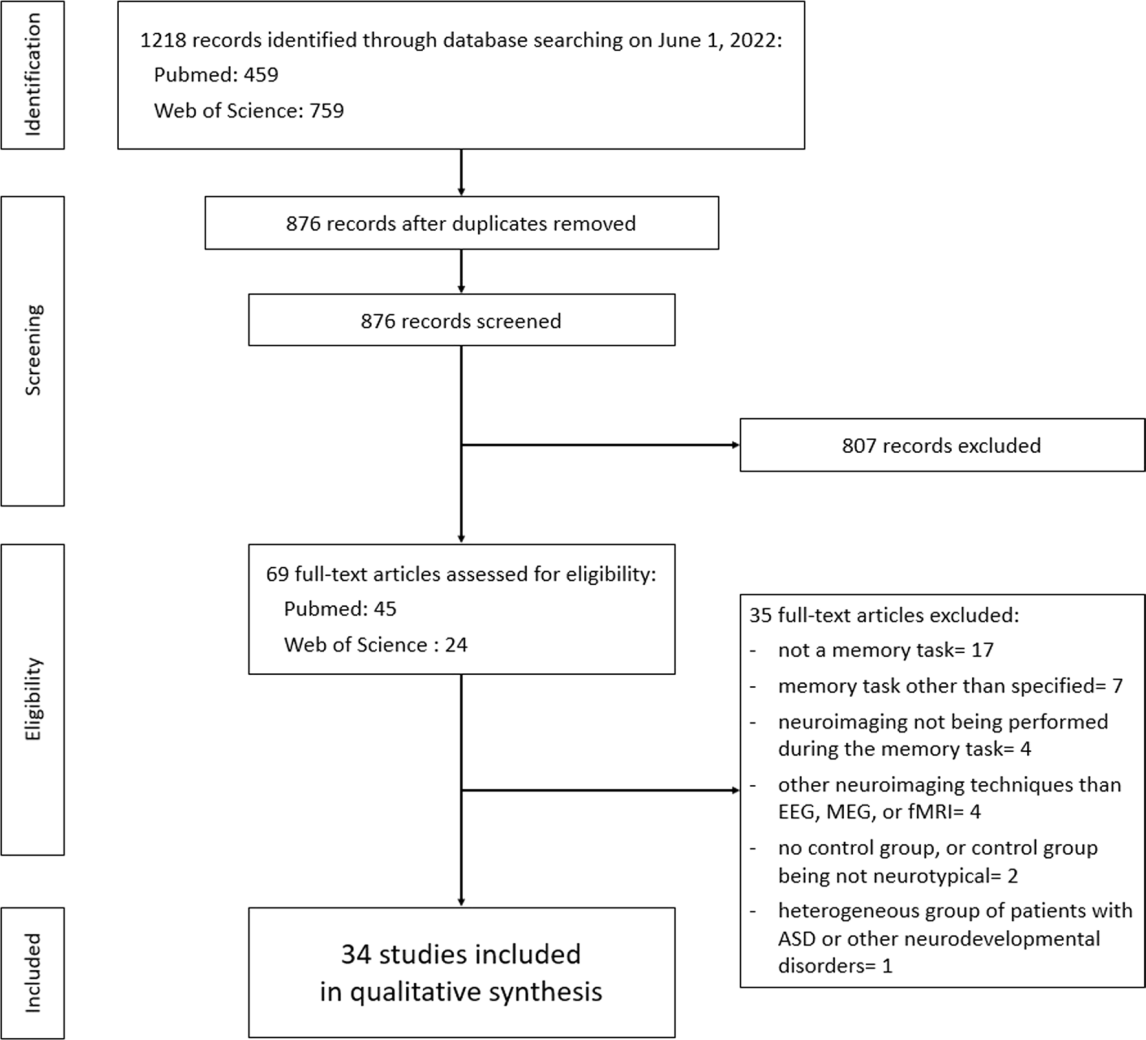
PRISMA (Preferred Reporting Items for Systematic Reviews and Meta-Analyses) flowchart describing the studies selection process

A literature search was conducted on *PubMed* and *Web of Science* databases on August 21, 2020, and updated on June 1, 2022, with no oldest limit date. Keywords used were both MeSH (Medical Subject Heading) and text word terms combined with Boolean terms: “(EEG OR MEG OR electroencephalography OR magnetoencephalography OR electrophysiology OR MRI OR Magnetic Resonance Imaging OR neuroimaging) AND (autism OR Asperger syndrome OR autistic OR pervasive developmental disorder) AND memory.” This led to 459 hits on *PubMed* and 759 hits on *Web of Science* (Fig. 1).

Limitation of bias was done at two levels. First, the first author (PD) screened independently all titles and abstracts, leading to 69 full-text articles assessed for eligibility. Inclusion of eligible studies was evaluated by two authors (PD and BG). Data extraction of included studies was conducted by the first author (PD), and a verification of extracted data was subsequently realized by two authors (PD and BG). A total of 35 studies were excluded for following reasons: no memory task (*n* = 17); memory task other than specified (e.g., learning) (*n* = 7); neuroimaging not being performed during the memory task (*n* = 4); other neuroimaging techniques than EEG, MEG or fMRI (e.g., tomography, functional near-infrared spectroscopy) (*n* = 4); absence of control group, or control group being not neurotypical (*n* = 2); and heterogeneous group of patients with ASD or other neurodevelopmental disorders (*n* = 1).

Finally, 34 articles were retained for synthesis, 19 focusing on WM, and 15 focusing on episodic LTM. Risk of bias analyses was also performed with regard to the quality of included studies.

Second, at the outcome level, two authors (PD and BG) independently evaluated the quality of included studies, using the *Standard Quality Assessment Criteria for Evaluating Primary Research Papers* [50]. The checklist was used in its original form, and all included studies were scored (2 = Yes, 1 = Partial, 0 = No, N/A = not applicable) with complete agreement between both authors. Assessment total scores were converted to a percentage score that ranged from 77 to 100%. A total of 33 studies met criteria for high-quality studies (Kmet score > 80%), among which 29 met criteria for very high quality (Kmet score > 90%); one study was evaluated of moderate quality (Kmet score: 77%). All studies were considered of sufficient quality (see Appendix).

## Results

### Studies on working memory

Nineteen WM studies were included [51–69] (Table 1), 16 of which used updating “N-back” tasks. It should be noted that N-back tasks may not fully reflect the manipulation of information by the central executive as theorized by the Baddeley model of WM [70], but rather the continuous updating of to-be-memorized information, which corresponds to storage without manipulation of information [71–73]. Included studies were informative either on the processing of to-be-memorized information only (*n* = 3), or on maintenance and manipulation of information only (*n* = 12), or both (*n* = 4), using fMRI for 15 studies, and electrophysiology for the other four ones.

**Table 1:** Included studies on working memory

Authors	N_ASD_/N_TD_	ASD age	Diagnosis method Diagnosis criteria Diagnosis	Material Memory task	Neuroimaging method Memory phase	Memory accuracy	Main results (ASD relative to TD groups) Conclusion
Audrain et al. [67]	24/30	28.2 ± 6.59	ADOS HFA	Visuospatial: abstract images 1-back	MEG Encoding, maintenance, recognition	Similar	Similar WM-related network in the theta frequency band between encoding and recognition and between ASD and TD, differing on maintenance Different WM-related network in the alpha frequency band between encoding, maintenance, and recognition and between ASD and TD Atypical functioning of WM maintenance, lack of inhibition of task-irrelevant processes
Barendse et al. [68]	13/13	15.3 ± 1.2	ADOS DSM-IV Autistic disorder, Asperger, PDD-NOS	Visuospatial: faces and houses 1- and 2-back	fMRI Recognition	Similar	Similar activity and functional connectivity Analyses of sparse matrices: reduced global efficiency suggesting an increase in network costs WM difficulties are compensated
Braden et al. [64]	16/17	50.1 ± 1.7	ADOS-2 DSM-IV DSM5 ASD	Verbal: letters 0-, 1-, 2-back	fMRI Recognition	Similar	Similar activation of the WM-related network, and similar deactivation of the DMN and salient network Decreased connectivity in the cortico-striatal-thalamic network when increasing memory load Functional brain difference persists with aging
Chantiluke et al. [59]	17/22	15.2 ± 1.8	ADI-R and ADOS ICD-10 ASD	Verbal: letters 1-, 2-, 3-back	fMRI Recognition	Reduced	Under placebo: underactivation of the right dorsolateral PFC, and enhanced DMN deactivation Under fluoxetine: normalization of the dorsolateral PFC activation, and sub-normalization of behavioral results Fluoxetine modulates WM brain activation
Hawco et al. [69]	24/20	22.5 ± 4.7	ADOS-2 ASD	Spatial: location of a dot 0-, 2-back	fMRI Recognition	Reduced, marginally significant	Greater variability in the pattern of dorsolateral PFC activation, in spite of similar between-group activation and connectivity Idiosyncratic prefrontal activation leads to diminished performance
Herrington et al. [55]	12/19	13.4 ± 4.2	ADI-R and ADOS DSM-IV Autism disorder, Asperger, PDD-NOS	Visuospatial: superimposed faces and houses 1-back	fMRI Recognition	Similar	When attending to faces: increased activation within multiple PFC areas, including dorsolateral part Suggests selective attention in order to further discriminate faces
Kleinhans et al. [57]	19/21	23.5 ± 7.8	ADI-R and ADOS DSM-IV Autistic disorder, Asperger, PDD-NOS	Visuospatial: neutral faces, or houses 1-back	fMRI Recognition	Similar	Similar activation of bilateral fusiform face areas, less functionally connected with both the subcortical face processing structures and the limbic structures Diminished connectivity within the limbic system supports social impairments
Koshino et al. [51]	14/13	25.7	ADI and ADOS HFA	Verbal: letters 0-, 1-, 2-back	fMRI Recognition	Similar	Reduced activation in the left hemisphere, enhanced activation in the right hemisphere and posterior regions Reduced frontal connectivity; enhanced connectivity within posterior/occipital areas Enhanced processing of low-level visual features
Koshino et al. [56]	11/11	24.5 ± 10.2	ADI-R and ADOS HFA	Visuospatial: neutral and emotional faces 0-, 1-, 2-back	fMRI Recognition	Similar	Reduced activations in both left inferior prefrontal areas and right posterior temporal areas Reduced fronto-fusiform functional connectivity Less verbally and socially oriented memory for faces
Larrain-Valenzuela et al. [66]	21/20	21.9 ± 4.1	AMSE DSM5 ASD	Verbal: consonants Modified Sternberg task	EEG Recognition	Reduced	Modulation of alpha power (occipital cortex) and theta power (left premotor cortex and the right PFC) in TD. Absence of load-dependent modulation of alpha and theta power in ASD Atypical oscillatory activity contributes to WM difficulties
Luna et al. [60]	11/6	32.3 ± 9.3	ADI-R and ADOS Autism	Visuospatial: location of a stimulus Oculomotor delayed response	fMRI Recall	Reduced	Decreased activation in both the dorsolateral PFC and the posterior cingulate cortex Fronto-parietal activation is reduced during WM
Rahko et al. [63]	28/22	14.6 [11.4–17.6]	ADI-R, ADOS, ASSQ ICD1-10, DSM-IV HFA, Asperger	Visuospatial: location of a stimulus 0-, 2-back	fMRI Recognition	Similar	Similar activation of WM-related areas, and similar DMN deactivation. Persistence of DMN when switching from 2- to 0-back in ASD only Inadequate memory load-dependent modulation of brain activity
Silk et al. [62]	7/9	14.7 ± 2.9	ADI DSM-IV Autistic disorder, Asperger	Visuospatial: 3-dimensional block shapes Mental rotation task	fMRI Recognition	Similar	Reduced activation in frontal cortical and subcortical structures. Similar parietal activation Dysfunction of the fronto-striatal network
Urbain et al. [54]	20/20	11.25 ± 1.58	ADOS HFA	Visuospatial: abstract images 1-, 2-back	MEG Recognition	Similar	Greater load-dependent activation in the left dorsal parietal cortex in the TD, while in the left ventral parietal cortex in ASD More controlled (i.e., top-down) processing of visual stimuli in the TD group, while being more automatic (i.e., bottom-up) in the ASD group
Urbain et al. [58]	17/20	11.17 ± 1.69	ADOS HFA	Visuospatial: abstract images 2-back	MEG Recognition	Reduced, marginally significant	Reduced fronto-temporal connectivity in the alpha band Reduced electrophysiological connectivity contributes to WM difficulties
Vogan et al. [52]	19/17	11.05 ± 1.43	ADOS HFA	Visuospatial: colored figures 1-back Color Matching Task, 6 levels of difficulty	fMRI Recognition	Reduced	Enhanced posterior activation. Reduced activation of frontal areas and the precuneus Less reliance on the fronto-parietal network for WM
Vogan et al. [61]	27/30	12.56 ± 1.46	ADOS ASD	Verbal: letters Letter matching task, 4 levels of difficulties	fMRI Recognition	Reduced	Reduced activation of fronto-parietal areas when increasing the memory load; similar DMN deactivation Inadequate modulation of fronto-parietal activity when increasing WM load
Vogan et al. [53]	14/15	13.4 ± 1.82	ADOS ASD	Visuospatial: colored figures 1-back Color Matching Task, 6 levels of difficulty	fMRI Recognition	Similar	2-years follow-up of Vogan et al. (2014) Minimal age-related changes in load-dependent activation, but greater longitudinal load-dependent deactivation in DMN Inadequate modulation of fronto-parietal activity when increasing WM load
Yuk et al. [65]	40/39	27.17 ± 6.27	ADOS ASD	Visuospatial: abstract images 1-, 2-back	MEG Maintenance, recognition	Similar	Maintenance: similar phase synchrony in theta, alpha, or beta bands; additional recruitment of fronto-parietal areas Recognition: diminished theta coherence between right frontal and left parietal areas Decreased theta connectivity; compensatory alpha recruitment

*ADI* Autism Diagnosis Interview; *ADI-R* Autism Diagnostic Interview-Revised; *ADOS* Autism Diagnostic Observation Schedule; *AMSE* Autism Mental Status Exam; *ASD* Autism Spectrum Disorder; *ASSQ* Autism Spectrum Screening Questionnaire; *DMN* (Default Mode Network); *DSM-IV* Diagnostic and Statistical Manual of Mental Disorders, 4th version; *DSM5* Diagnostic and Statistical Manual of Mental Disorders, 5th version; *ICD-10* International Classification of Diseases, 10th version; *PFC* prefrontal cortex; *TD* Typically Developing); *WM* Working Memory

#### Effects of the type of material on neuroimaging results

Only one study focused on WM processing of visually presented verbal information, using letters. Other reviewed studies concerned mostly visuospatial information (e.g., colored figures, abstract images, or stimulus location), including faces. Most of these studies reported similar memory performance (thereafter, “performance”) between ASD and TD participants, with different patterns of brain activation and connectivity.

Using an fMRI letter N-back task, Koshino et al. [51] identified in the 2-back condition, a pattern of activation and connectivity being lateralized right or bilateral posterior in adults with ASD relative to TD, with reduced activations in left fronto-parietal areas, also in participants with ASD relative to TD. These results suggest a more visual graphical, i.e., less semantic, processing of letters, in spite of similar between-group performance.

Other studies used visuospatial material. Vogan et al. [52] conducted an fMRI 1-back color matching task in preadolescent children and observed that when the number of different colors constituting a figure increased, ASD relative to TD children showed diminished performance and diminished parietal activation while showing similar activations in bilateral occipital areas and fusiform gyri. In a 2-year follow-up study, Vogan et al. [53] observed in the same participants, a greater load-dependent activation in parietal lobes only in TD controls. The minimal changes they observed in the ASD group were limited to occipito-temporal areas, with diminished performance on the higher load conditions. Results from these two studies suggest preserved activation within the visual occipito-temporal areas associated with the “ventral stream,” while an under-recruitment of parietal areas associated with the “dorsal stream,” with a limited integration of these two streams during visual processing over time. Close to these results, Urbain et al. [54] used a visual N-back task with MEG in children and identified a greater load-dependent activation in the left dorsal parietal cortex in the TD children, while in the left ventral parietal cortex in those with ASD. Despite similar performance, this distinction would reflect a more controlled (i.e., top-down) processing of visual stimuli in the TD group, while being more automatic (i.e., bottom-up) in the ASD participants, in line with the distinction between dorsal and ventral attentional systems within the parietal cortex [74]. In summary, these studies suggest that visual processing during WM tasks is associated with increased activations in ventral areas alongside diminished activation in dorsal areas, in individuals with ASD relative to TD.

Three fMRI studies have documented atypical processing of faces in WM in ASD, reporting similar performance between ASD and TD groups. Herrington et al. [55] conducted a 1-back task in children using superimposed images of neutral or emotional faces and houses, with targets containing either same or different faces or houses. They observed a similar activation of the fusiform gyrus in both groups. By contrast, increased activation of the dorsolateral prefrontal cortex was found in the ASD group when the target contained a face, suggesting selective attention in order to further discriminate faces. For the TD group, increased activation was found when the target contained a house, suggesting selective attention in this group was aimed at disregarding the automatic percept of a face. Koshino et al. [56] used an N-back task with mixed neutral and emotional faces, in adolescents and young adults and observed reduced activations in both left inferior prefrontal areas usually associated with verbal processing and right posterior temporal areas usually associated with theory of mind, in participants with ASD compared with TD. Activations within the bilateral fusiform face areas (FFA), a key structure enabling the processing of basic facial features and also involved in object identification [75], were similar but atypically located in ASD relative to TD (i.e., location of peak activation differed from that of the TD group), and also less connected to frontal areas. In adults, Kleinhans et al. [57] used a 1-back task with neutral faces and reported similar activation of bilateral FFA in ASD and TD groups, but was less functionally connected in ASD with both the subcortical face processing structures (amygdala and superior colliculi) associated with fast emotional attention for faces [76] and the limbic structures (precuneus and posterior cingulate gyri) that enable the processing of emotional stimuli [77]. Overall, these results point toward ASD participants having a relatively similar level of FFA activation, but lower activity and connectivity with brain areas related to the attention to faces and their socio-emotional processing.

#### Short-term maintenance and manipulation

Included studies reported reduced activation of prefrontal areas (*n* = 9), reduced deactivation of the DMN (*n* = 6), and atypical functioning of the WM-related brain network (*n* = 7), mainly during WM maintenance.

First, just over half (i.e., 9/16) of fMRI N-back studies found reduced prefrontal activations in ASD relative to TD, associated with diminished or enhanced activations within posterior areas. In studies reporting reduced prefrontal activations in conjunction with enhanced parietal or temporal activation, performance was similar between ASD and TD groups in children and adults [51, 53, 56, 58]. By contrast, other fMRI studies that reported reduced prefrontal activations without enhanced parietal or temporal activations found significantly diminished performance in groups with ASD relative to TD, in children and adults [52, 59–61]. Close to these findings, in a short report using an fMRI visuospatial mental rotation task requiring manipulation of information, Silk et al. [62] found in adolescents with ASD relative to TD diminished activations within cortical and subcortical (caudate head) frontal regions, with similar parietal activation, while similar between-group performance. Taken together, these results suggest a general, diminished load-dependent recruitment of the prefrontal areas, being partially or fully offset by enhanced activation in posterior sensory and attentional areas.

Second, some studies investigated the DMN, reporting atypical deactivation in the higher load conditions of fMRI N-back tasks in ASD, with similar performance in the ASD and TD groups. In adolescents with ASD relative to TD, Chantiluke et al. [59] found in the verbal 3-back condition, reduced activation of the dorsolateral prefrontal cortex associated with enhanced DMN deactivation, suggesting a compensatory process. In adolescents too, Rahko et al. [63] observed similar activation of WM areas in the visuospatial 2-back condition and similar DMN deactivation in ASD and TD groups. This persisted in the 0-back condition in the ASD group only and was associated with diminished performance. In contrast to these results, Vogan et al. [52, 61] found a similar DMN deactivation in a visual 1-back task with different levels of difficulty in children and adolescents with ASD and TD, suggesting no reduced load-dependent modulation of DMN deactivation. However, in a 2-year follow-up study with the same participants, Vogan et al. [53] reported a greater longitudinal load-dependent DMN deactivation in those with ASD compared with TD. In middle-aged adults with and without ASD, Braden et al. [64] identified with a verbal 0-, 1-, and 2-back task, similar load-dependent DMN deactivation and similar performance between groups. Hence, there are no clear results about DMN deactivation during WM tasks in ASD.

Third, more recent studies have explored memory networks in ASD focusing on slow frequency bands in EEG/MEG. Urbain et al. [58] identified reduced fronto-temporal alpha connectivity associated with diminished performance during a visual 2-back condition in children with ASD relative to TD. This points to a less efficient WM network during maintenance. Using the same paradigm in young and middle-aged adults, Yuk et al. [65] found similar performance across ASD and groups TD, but connectivity results differed for each phase. Maintenance was associated with an alpha connectivity network common to both groups, with an additional recruitment of fronto-parietal areas and increased coherence, and recognition was associated with diminished theta coherence between right frontal and left parietal areas, when comparing groups with ASD and TD. In adults too, Larrain-Valenzuela et al. [66] manipulated memory load during a modified Sternberg task and found that for TD participants, there was enhanced alpha power in occipital areas and enhanced theta power in bilateral frontal areas when load increased. There was, however, no load-dependent modulation of alpha and theta power in the ASD group, indicating that atypical oscillatory activity in ASD may contribute to diminished WM performance. More recently, Audrain et al. [67] identified in a visual 1-back task in adults with ASD and TD with similar performance, that connectivity in the theta band during maintenance phase consisted mainly in interhemispheric posterior connections in ASD, while being mainly antero-posterior in TD. In addition, connectivity in the alpha band differed in ASD and TD in all WM phases. These results point toward a more atypically functioning WM maintenance relative to encoding and retrieval stages in ASD (theta band), but a global lack of inhibition of task-irrelevant processes (alpha band). Overall, in spite of contrasting results, a decrease in antero-posterior theta connectivity seems to characterize the WM maintenance phase in autistic individuals. Enhanced recruitment of the alpha band may correspond to a compensatory process, enabling greater inhibition to avoid interference.

Other studies on WM networks used fMRI during N-back tasks. In middle-aged adults, Braden et al. [64] identified a similar cortico-striatal–thalamic–cortical network of activation in ASD and TD groups but a reduced functional connectivity within this network in ASD alongside similar performance, when switching from 0- to 2-back condition. Using the method of connectivity matrices, Barendse et al. [68] identified a lower global efficiency (*i*.*e*., diminished transmission of neural information within the network in adolescents with ASD) along with performance in normal range. More recently, Hawco et al. [69] reported within-group differences in the location of prefrontal activations in young adults with ASD, associated with a trend for reduced performance when compared with TD participants, suggesting a link between idiosyncratic prefrontal activation during WM and diminished performance.

### Studies on episodic long-term memory

Fifteen studies were included [78–92] (Table 2), which were informative either on the processing of memorized information only (*n* = 6), or on memory-related processes during either encoding or retrieval phases only (*n* = 7), or both (*n* = 1), using fMRI or electrophysiological methods.

**Table 2:** Included studies on episodic long-term memory

Authors	N_ASD_/N_TD_	ASD age	Diagnosis method Diagnosis criteria Diagnosis	Material Memory task	Neuroimaging method Memory phase	Memory accuracy	Main results (ASD relative to TD groups) Conclusion
Chan et al. [86]	21/21	10.27 ± 2.26	DSM-IV Autistic disorder, PDD-NOS	Visuospatial: line drawings Object recognition task	EEG Recognition	Reduced	Greater antero-posterior theta connectivity in the left hemisphere in ASD, and in the right hemisphere in TD. Negative correlation between coherence and performance in ASD only Threshold of connectivity beyond which performance decreased
Churches et al. [81]	11/11	31.82 ± 6.88	DSM-IV Asperger	Visuospatial: neutral faces Recognition	EEG Recognition	Reduced	Reduced amplitudes on the N170 potential for both target and non-target faces; reduced amplitude on the N250 potentials to target faces only The development of new face representation is impaired
Cook et al. [92]	12/19	13.18 ± 1.73	ADI-R and/or ADOS DSM5 ASD	Visuospatial: object–scene pairs Rate the congruence	fMRI Encoding	Reduced object recognition Similar associative recognition	TD: encoding of subsequently recognized pairs associated with greater activation of the bilateral medial prefrontal cortex for intermediate relative to congruent pairs, and with greater activation of the left medial temporal lobe for congruent relative to intermediate pairs ASD: same level of activation regardless of the level of congruence; higher activations in bilateral, prevailing left medial prefrontal areas, for participant with greater behavioral flexibility Lack of modulation of encoding-related processes with the level of congruency
Cooper et al. [90]	24/24	30.3 ± 8.4	DSM5 or ICD-10 ASD	Visuospatial: background, and objects Recognition	fMRI Encoding and recognition	Reduced	Encoding: similar levels of activity and connectivity among prefrontal, hippocampal and parietal areas Recognition: limited differences: reduced left prefrontal activity, and reduced hippocampal connectivity with the fronto-parietal areas Memory representations are processed similarly by the hippocampus; limited pre-retrieval search or post-retrieval monitoring
Desaunay et al., [79]	22/32	16.51 ± 3.56	ADI-R and/or ADOS DSM5 and ICD-10 ASD	Visuospatial: picture pairs Associative recognition	EEG Recognition	Reduced	Reduced amplitude on the P2 and FN400 potentials: limited integration of low-level perceptual into high-level conceptual information, decrease in familiarity strength Spatial extension of the LPC: suggests additional recruitment of associative processes Effortful retrieval of associative information compensates for the lower familiarity strength
Gaigg, et al. [89]	13/12	35.6 ± 10.3	ADOS DSM-IV ASD	Verbal: word triplets, semantically related or not Associative recognition	fMRI Encoding	Similar	Similar brain activations, including the left inferior frontal gyrus and the left hippocampus, with similar signal change with the degree of relational encoding Only limited signal change in prefrontal and posterior hippocampal regions, when contrasting the degree of awareness for subsequent retrieval Attenuated levels of recollection are related to anomalies in relational encoding processes
Greimel et al., [82]	13/13	15.9 ± 3.0	ADI-R and ADOS DSM-IV and ICD-10 HFA, PDD-NOS, Asperger	Visuospatial: objects superimposed with faces or houses Recognition of the objects	fMRI Encoding	Similar	Encoding of subsequently recollected objects presented with a face: reduced activations within bilateral inferior and medial frontal gyri and the right intraparietal lobule Encoding of subsequently non-recollected objects presented with houses: increased activation of the dorsal-medial PFC Incidental encoding of faces in the ASD group is less automatic and less associated with social information
Gunji et al. [80]	8/9	10.8 ± 2.9	DSM-IV HFA, Asperger	Visuospatial: self, familiar, unfamiliar faces Recognition of the subject’s own face and familiar faces	EEG Recognition	Similar	Shorter N170 latency No amplitude difference on the early posterior negativity between Self vs. Familiar and Familiar vs. Unfamiliar No amplitude difference on the P300 between familiar and unfamiliar faces Deficit of semantic encoding of faces
Hogeveen et al. [91]	47/60	18.4 ± 2.81	ADOS-2 DSM5 ASD	Visuospatial: item-specific or relational encoding Item or associative recognition	fMRI Encoding	Similar (for item and associative recognition)	Increased hippocampal activity along with diminished connectivity between the medial temporal lobe and postero-medial brain regions Hippocampal hyper-recruitment suggests compensation for the decrease in regional connectivity to support the preservation of associative memory
Lynn et al. [83]	13/13 15/16 13/14	11.7 ± 1.2 15.3 ± 1.8 24.9 ± 4.9	ADI and ADOS Autism	Visuospatial: faces and cars Recognition	fMRI Encoding and recognition	Reduced (for both faces and cars)	Encoding and recognition: face-specific underconnectivity between bilateral fusiform face areas and the medial PFC Recognition: both general (i.e., cars) and specific (i.e., faces) underconnectivity between the right fusiform face area and the visual cortex Diminished integration of information; abnormalities in perceptual systems also contribute to recognition deficits, especially with faces
Massand et al. [87]	22/14	25.72 ± 4.76	ADI-R and/or ADOS DSM-IV ASD	Verbal: high- and low-frequency words Recognition	EEG Recognition	Similar	Parietal rather than anterior early FN400 old/new effect related to familiarity Similar parietal LPC potential related to recollection Preservation of the electrophysiological recollection process
Massand & Bowler [88]	15/18	38.89 ± 14.77	ADOS DSM-IV Autism, Asperger	Visuospatial: line drawings of objects Recognition (line drawings) and recall (their color)	EEG Recognition and recall	Reduced recognition	Posterior and attenuated FN400 old/new effect related to familiarity, occurring in a large time-window (300-650 ms) Similar parietal LPC potential related to recollection Preservation of the electrophysiological recollection process
Neumann et al. [78]	7/7	20.9 ± 10.6	ADI-R ICD-10 HFA “mnemonist savants”	Verbal: pseudowords Visuospatial: abstract shapes Recognition	MEG Recognition	Reduced (pseudowords) Similar (shapes)	Both stimuli: early right occipital activation (100-200 ms) in ASD only Shapes: bilateral old/new effect (200-500 ms) reflecting familiarity-based recognition, being more left hemispheric in controls Expertise with figural material; pseudowords were processed and recognized similarly to visual material
Noonan et al. [85]	10/10	23 ± 9.9	ADI-R and ADOS DSM-IV HFA	Verbal: words read or heard Recognition (item and source)	fMRI Recognition	Reduced	Unexpected enhanced left fronto-parietal functional connectivity Atypical functional connectivity
O’Hearn et al. [84]	15/13 16/19 14/15	11.7 ± 1.17 15.39 ± 1.17 24.56 ± 4.87	ADI and ADOS Autism	Visuospatial: faces and cars Recognition	fMRI Encoding and recognition	Reduced (for both faces and cars)	Similarity of fusiform face area activation increased from childhood to adolescence, then decreased from adolescence to adulthood Recognition performance was more related to the similarity score in the left fusiform face area Reduced specialization of the fusiform face area

*ADI* Autism Diagnosis Interview; *ADI-R* Autism Diagnostic Interview-Revised; *ADOS* Autism Diagnostic Observation Schedule; *AMSE* Autism Mental Status Exam; *ASD* Autism Spectrum Disorder; *ASSQ* Autism Spectrum Screening Questionnaire; *DMN* (Default Mode Network); *DSM-IV* Diagnostic and Statistical Manual of Mental Disorders, 4th version; *DSM5* Diagnostic and Statistical Manual of Mental Disorders, 5th version; *ICD-10* International Classification of Diseases, 10th version; *LTM* Long-term memory; *PFC* prefrontal cortex; *TD* Typically Developing)

#### Effects of the type of material on neuroimaging results

Only one study focused on visually presented verbal information. The studies reviewed used mainly visuospatial material including faces. We first describe the electrophysiological and then the fMRI results.

Neumann et al. [78] were interested in the old/new effects with MEG elicited by the recognition of pseudowords or abstract shapes. The memory performance of the participants with ASD was lower than TD for the former type of stimuli and similar for the latter. The authors identified an early right occipital activation (100–200 ms) in ASD with both kinds of stimuli that did not occur in controls, followed by a bilateral old/new effect (200–500 ms) with shapes in ASD participants reflecting familiarity-based recognition that was more left hemispheric in controls. These results suggest that pseudowords were processed and recognized similarly to visual material in ASD relative to TD participants.

Desaunay et al. [79] used a visual associative recognition task and identified a similar ERP time-course on the occipital P2 (220–270 ms), mid-central FN400 (350–470 ms), and parietal LPC (600–700 ms) potentials in adolescents and young ASD and TD adults, whereas performance for ASD participants was lower. Amplitudes were reduced in the group with ASD relative to TD on both the P2 potential, thought to index an intermediate processing stage linking elementary perceptual processes with higher-level semantic processes [93], and the FN400 potential, that reflects both conceptual priming and familiarity process [94]. Two ERP studies on face recognition reported similar performance between ASD and TD, but differences on both the temporal N170 potential (peaking 170 ms, and indexing attention and structural perception of faces), and the temporal N250 potential (also labeled as EPN, peaking 250 ms, and indexing familiarity-based recognition for faces [95]). In children, Gunji et al. [80] found a shorter latency on the N170 potential for self, familiar and unfamiliar faces in ASD relative to TD. Churches et al. [81] investigated the recognition of unfamiliar faces after a study phase and found reduced amplitudes of the N170 and N250 potentials in participants with ASD relative to TD. In summary, these amplitude decrements suggest reduced attention to visual stimuli and reduced integration of low-level perceptual into high-level conceptual information and the semantic memory system, with a decrease in familiarity strength during visual recognition in ASD.

Other studies used fMRI. Greimel et al. [82] studied in children and adolescents, the encoding of visually presented objects superimposed with either faces or houses. Despite similar performance at test, encoding of subsequently recognized objects presented with a face was associated, in ASD relative to TD, with reduced activations within bilateral inferior and medial frontal gyri and the right intraparietal lobule, suggesting that encoding of faces in the ASD group was less automatic and less associated with social information than in TD. Recently, Lynn et al. [83] evaluated the functional connectivity of the FFA for faces versus non-faces (cars) stimuli, at encoding and recognition stages in children, adolescents, and adults. TD participants tended to improve performance from adolescence to adulthood, while those with ASD did not. With faces, the authors found reduced connectivity between bilateral FFA with both prefrontal and primary visual cortices in ASD relative to TD. This was independent of age and occurred at both stages, suggesting that reduced integration of information between distributed brain areas may lead to weaker representation of faces in memory. These authors also identified in ASD, age-related reduced connectivity between the right FFA and the visual cortex only during the recognition phase, suggesting that memory difficulties with faces may arise more during recognition than encoding. Extending these results in an updated study, the same team investigated the age-related changes in the similarity of FFA activation [84]. For TD participants, they found an increasing overlap of activations within a category (faces, cars) from adolescence to adulthood in the right FFA—that processes faces holistically while the left FFA processes them more featurally [96]—reflecting brain maturation for holistic processing. In ASD. Similarity of FFA activation increased from childhood to adolescence, presumably reflecting maturation, but then decreased from adolescence to adulthood. Recognition performance was more related to the similarity score in the left FFA—while in the right FFA in TD—which together suggests reduced FFA specialization for faces and object identification in ASD, and a more featural rather than configural processing of these stimuli. Thus, fMRI studies complement those in EEG, by showing diminished FFA specialization for faces and objects and reduced integration of information between distributed brain areas including bilateral FFA and the visual cortex.

#### Encoding and retrieval processes in episodic LTM

Studies focusing on encoding and retrieval processes in LTM explored either the functional connectivity during single item recognition (*n* = 2), the electrophysiological recollective old/new effect (*n* = 3), or associative processes with fMRI (*n* = 4).

First, Noonan et al. [85] conducted an fMRI source recognition paradigm in adolescents and adults using visually presented target words previously presented in a visual or auditory modality. Functional connectivity was analyzed between three seed regions of interest (left middle frontal, left superior parietal, and left middle occipital cortex) and the whole brain. The authors found, in spite of diminished performance in ASD, a large pattern of similar connectivity in ASD and TD groups and unexpectedly enhanced left fronto-parietal functional connectivity in participants with ASD relative to TD. Close to these results, Chan et al. [86] investigated in children and adults, the coherence in the theta band—associated with WM and LTM memory processes—during the recognition phase of previously seen pictures. Antero-posterior theta connectivity was greater in the left hemisphere in ASD participants and in the right hemisphere for TD ones, with a negative correlation between coherence and performance in the ASD group only, suggesting a threshold of connectivity beyond which performance decreased. Taken together, these two studies identified enhanced hemispheric left connectivity during episodic recognition in the ASD compared with the TD participants.

Using ERPs, Massand et al. [87] and Massand and Bowler [88] conducted two successive studies focusing on the familiarity-related FN400 and recollection-related LPC potentials; overall memory performance was similar in ASD and TD groups in both studies. Massand et al. [87] employed a single-word recognition paradigm and found a parietal rather than anterior early FN400 old/new effect (300–500 ms) in adults with ASD relative to TD, followed by a similar parietal LPC recollective process (500–800 ms) in both groups. Massand and Bowler [88] used a single-picture recognition test followed by a phase where the color of the studied items had to be recalled. The authors found a posterior and attenuated FN400 old/new effect in the ASD group, occurring in a large time-window (300–650 ms), followed by a similar LPC old/new effect. More recently, Desaunay et al. [79] used a visual associative recognition task in a study of adolescents with ASD. They found in conjunction with diminished performance in the ASD relative to the TD participants, a reduced FN400 amplitude followed by a LPC old/new effect presenting a parietal extension. These findings suggest that effortful retrieval of associative information compensates for the lower familiarity strength in the ASD participants. Overall, results from these three ERP studies indicate that recognition in ASD is qualitatively similar to that seen in TD and relies on the same dual-process, but may, however, differ quantitatively, with diminished familiarity-related potentials and preserved or even enhanced recollection-related potential in ASD.

Other fMRI studies explored associative memory in ASD, with a focus on hippocampus as a key structure underlying inter-item and item-context associations [97]. Gaigg et al. [89] evidenced a strong overlap of brain activations in adults with ASD and TD—including the left inferior frontal gyrus and left hippocampus—with similar signal change with the degree of relational encoding of word triplets accompanied by diminished subsequent recognition. Between-group difference was limited to absent or reduced signal change in prefrontal and posterior hippocampal regions in the ASD group relative to TD, when contrasting the degree of awareness (familiarity/recollection) for subsequent retrieval. Three recent studies have investigated associative memory with visual material. First, using a paradigm designed to assess memory precision, Cooper et al. [90] identified similar levels of activity and functional connectivity among prefrontal, hippocampal and parietal areas during encoding, in adults with ASD and TD. By contrast, the recognition phase included several differences albeit close performance, including reduced left prefrontal activity, suggesting limited pre-retrieval search or post-retrieval monitoring, and reduced hippocampal connectivity with the fronto-parietal network. Second, Hogeveen et al. [91] investigated the associative encoding of paired pictures in adolescents and young adults and identified increased hippocampal activity along with diminished connectivity between the medial temporal lobe and postero-medial brain regions in the ASD relative to the TD group, alongside similar between-group performance. This enhanced activity and reduced connectivity were significantly inversely correlated, indicating that the decreased inter-regional connectivity in ASD would be efficiently compensated for by hippocampal hyper-recruitment to support preserved associative memory. Fronto-hippocampal connectivity was also reduced in the ASD group compared with TD group. Third, Cook et al. [92] performed an associative encoding task, asking participants from late childhood to adolescence to rate if object–scene pairs were congruent, incongruent, or intermediate. Participants with ASD and TD showed both a similar congruency rating during encoding, and the same facilitation for better subsequently recognizing congruent than incongruent pairs. In the TD group, encoding of subsequently recognized pairs was associated with greater activation of the bilateral medial prefrontal cortex for intermediate relative to congruent pairs, and with greater activation of the left medial temporal lobe (including the hippocampus) for congruent relative to intermediate pairs. By contrast, activations of these two regions did not vary according to the level of congruency in the ASD group. As congruent information encoding is progressively supported by the medial prefrontal cortex throughout development, the lack of modulation observed in the ASD group may reflect developmental immaturity; participant with ASD with greater behavioral flexibility had higher activations in bilateral, prevailing left medial prefrontal areas. Taken together, these fMRI results extend those in EEG, showing that processing of associative information is qualitatively similar in ASD as in TD. Notably, hippocampal activation is similar or enhanced in ASD compared with TD; between-group differences mainly consist in reduced prefrontal activation and hippocampal connectivity.

## Discussion

This systematic review highlights both great differences and similarities in memory functioning between ASD and TD, depending on the type of material (verbal, visuospatial, faces), the type of memory (WM or episodic LTM), and the memory phase examined (mainly episodic encoding and retrieval). We attempt to relate these observations to the patterns of presence or absence of memory difficulties in ASD that have been identified in several meta-analyses. We also attempt to advance hypotheses based on neuroimaging findings relating to the specific characteristics of memory processes and representations in ASD.

### Hemispheric asymmetries: stronger representation of verbal than visuospatial information

Meta-analyses about memory functioning in ASD suggest lower WM and episodic LTM performances for visuospatial than verbal material, and even larger memory difficulties for spatial memory and face recognition [5, 44, 46].

For verbal material, atypical right-hemisphere activity was identified during WM tasks using letters [51] and LTM tasks using pseudowords [78]. Such findings suggest that these kinds of stimuli orient participants with ASD toward a shallower processing because of their tendency to process verbal information in a less semantic way than TD when not encouraged by the task [98]. Interestingly, encoding of visually presented words was associated with similar activations and enhanced connectivity within the left hemisphere in ASD relative to TD [85, 89], which suggests that brain functioning in ASD is closer to TD when the memory task material typically triggers left-hemisphere processing. This could be supported by a tendency for the left hemisphere to be less atypically developed than the right, as highlighted by recent anatomical and functional connectivity studies in ASD [99–101]. This relative preservation of hemispheric left over right connectivity, in conjunction with the typical left-hemisphere specialization for semantic memory and language [102–104], could result in better memory for verbal over other types of material in ASD.

The autism-specific characteristics of visuospatial memory could also be related to the relative preservation of hemispheric left over right connectivity. Two studies found that the left FFA, when compared with right FFA, has a greater maturation [83] and a less reduced connectivity with the visual cortex [84] in ASD. Two other studies reported bilateral, prevailing left medial prefrontal areas activations during picture encoding [92], and a leftward lateralization of functional connectivity during picture recognition [86]. Less atypical connectivity in the left than in the right hemisphere in ASD has been particularly demonstrated for intra-hemispheric visual-association fibers [105], especially for the inferior longitudinal fasciculus supporting visual processing [106]. This asymmetry of hemispheric connectivity, in conjunction with both hemispheric visual specialization (*i*.*e*., local-featural and global-configural processing being supported by the left and right hemispheres, respectively [107]) and reduced interhemispheric transfer of information due to underconnectivity [99, 108], could lead to a more featural memory for visual items, less related to their long-term conceptual (semantic) representations. In this sense, hemispheric isolation with reduced local/global integration of visual information has been evidenced during a visual categorization task with EEG [109].

Functional imbalance between ventral and dorsal streams may also account for visuospatial difficulties in ASD [110, 111]. Indeed, some studies have identified similar activations within the ventral stream in ASD as in TD while being diminished within the dorsal stream [52–54], which corroborates the neuroimaging account of a specific dysfunction of the dorsal stream in visual perception in ASD [112]. The ventral stream is typically associated with object recognition, while the dorsal stream is most selective for spatial information and direction of movement. Dorsal-to-ventral transfer of information continually adjusts ventral stream processing toward the most identification-relevant features of an object [113], facilitating face and object recognition [114]. Hence, atypical dorsal stream functioning, possibly resulting from decreased anatomical connectivity of the superior longitudinal fasciculus and uncinate fasciculus [112], which are among the most commonly reported association fibers with altered white matter integrity in ASD [108], may reduce the processing of intra-item spatial information, also accounting for the weaker representation of visuospatial information in ASD. The maturation of the visual system in TD individuals associates prolonged gray matter development within the ventral stream, supporting its functional specialization, but later development of white matter connectivity within the dorsal stream, that improves higher-order visual perception over time [115]. Hence, atypical FFA specialization and dorsal stream activation identified in the studies reviewed above may reflect developmental differences between ASD and TD.

Similar atypical processes may also account for face memory difficulties in ASD, since face recognition typically implicates the configural processing of facial features as in a spatial task [116]. In that sense, the typical rightward lateralization of the N170 potential is generally attenuated in ASD [117]—although this was not investigated in the studies reviewed above. A similar level of activation was observed in the FFA in ASD as in TD in some studies [55, 57, 84], which suggests that the processing of basic facial features is preserved, but with a lower specialization for this kind of stimuli [84]. By contrast, a global decrease in FFA connectivity suggests that the typical integration of multimodal information in memory for faces is reduced in ASD, including perceptual visual information within the ventral stream [83, 84], and socio-emotional information [56, 57], along with a less automatic attention to these stimuli [55, 82].

Taken together, neuroimaging studies suggests that the processing of written words during memory tasks is relatively similar in ASD as in TD, while the conceptual processing of visuospatial information including faces is reduced. Words would be more associated with their long-term representation in the semantic memory system than visuospatial information including faces. Indeed, cognitive models suggest that efficiency of encoding, storage, and retrieval, for either WM or LTM, is related to the level of interaction between information processing and the semantic memory system [10, 118]. This causal link between reduced conceptual processing of visuospatial information and weaker representation in memory has been demonstrated with EEG studies showing amplitude decrements on the early potentials associated with priming and next on the familiarity-related potential [79–81]. Familiarity, a graded signal that increases with the number of intra-item informational links that match the specific face or object stored into the semantic memory system [119] is then subsequently reduced. This account is borne out by a behavioral study reporting that memory for semantically related pictures in ASD is enhanced by associating picture names to the pictures themselves, suggesting that words would foster item and inter-item conceptual processing, leading to better memory [120].

### Antero-posterior asymmetries: high-fidelity WM representations with few top-down processes

Most meta-analyses have reported a medium or medium to large effect sizes for ASD-TD differences in WM [5, 43, 44, 121], indicating autism-related difficulties with active storage and manipulation of information.

Diminished load-dependent activation of prefrontal areas, without or with compensatory enhanced temporo-parietal activation, was the most consistent finding in the WM studies in ASD reviewed here. It may implicate the reduced long-range functional connectivity in ASD, which results in a lower recruitment of anterior compared with posterior brain areas [108, 122]. In that sense, one fMRI study reported a decrease in functional connectivity among a large brain network [64], and several EEG/MEG studies reported a decrease in antero-posterior alpha and theta coherence during the maintenance phase [58, 65, 67], in accordance with the long-range underconnectivity at lower frequency bands in ASD [100]. Alternatively, reduced load-dependent prefrontal areas, in conjunction with reduced parietal activation [52, 53, 61, 63], may implicate a general lack of modulation with memory tasks demand in ASD [123], or the general idiosyncratic brain activation in ASD [124, 125], as shown in prefrontal areas [69].

The second main result was the similar or enhanced DMN deactivation in ASD compared with TD. Enhanced DMN deactivation found in adolescents with ASD [53, 59] may correspond to a compensatory process for the under-recruitment of prefrontal areas during short-term maintenance, since DMN deactivation typically correlates negatively with fronto-parietal activation during WM [18] and predicts memory performance [126]. On the other hand, in the context of a developmental shift in TD from DMN hyper-connectivity in childhood to underconnectivity in adolescence and adulthood, this increased DMN deactivation may result from the delayed maturation of DMN in adolescents with ASD [127, 128]. This lack of coherence among results prevents any clear conclusions on the role of DMN functioning in WM in ASD.

Recent research in TD individuals suggests that high-fidelity storage of WM representations strongly involves sensory cortices [129]. By contrast, as neurons in the prefrontal cortex have been shown to be involved in the coding for combinations of item- and task-related information [130], subsequent studies have convergingly concluded that persistent prefrontal activity during WM maintenance is associated not only with rehearsal, but also with selective task-related information, providing top-down control to select stimulus-specific activation in sensory regions [131]. WM tasks with low information processing demand would mainly rely on sensory cortices, while those requiring higher abstraction or information processing would necessitate enhanced prefrontal top-down control. In addition, three of the reviewed WM studies reported diminished fronto-striatal connectivity in ASD relative to TD [58, 62, 64] that is typically associated with WM updating [132–134]. Information transferred from the striatum to the prefrontal cortex is related to the task set, controlling when representation within the prefrontal cortex should be maintained as opposed to updated, e.g., during manipulation or to avoid interference. Hence, reduced prefrontal activation and striato-prefrontal connectivity in ASD may suggest diminished rehearsal and top-down processes, which in turn may impair active maintenance of information and make it sensitive to decay. Representation of WM information in posterior temporo-parietal areas may be similar in ASD as in TD, but less specifically relevant to the task set, and more sensitive to interference.

Though typical WM development involves than same—primarily fronto-parietal—brain areas over time, age-related activations increase in frontal areas and decrease in posterior areas [135, 136]. To some extent, the WM network in ASD may result from immature development due to altered integrity of frontal white mater or long-range fibers. On the one hand, anatomical connectivity in ASD is more reduced in regions that include frontal lobe pathways compared to other brain regions [137]. On the other hand, the superior longitudinal fasciculus and occipitofrontal fasciculi are two of the most commonly reported association fibers with altered white matter integrity in ASD [108], while their preserved integrity correlates with WM performance in TD [138].

### Preserved posterior and hippocampal activity: weak context processing does not impair relational information in episodic LTM

A meta-analysis on memory in ASD reported a small to medium effect size in episodic LTM, suggesting difficulties at encoding and retrieval stages of memory processing [5]. Associative memory was not any more diminished than non-associative memory in subgroup comparisons. Most of the studies reviewed here reported asymmetries between left and right hemispheres, posterior and anterior areas in ASD, when compared with TD. Although findings from episodic memory studies are heterogeneous, they are also consistent with these differences.

First, the results are consistent with the account of preserved memory functioning in ASD for tasks involving more left-hemisphere neurophysiological processes than right ones, such as verbal memory tasks [85, 89]—in line with the relative preservation of hemispheric left over right connectivity in ASD [99–101]. Besides, the leftward lateralization of medial prefrontal areas activations during picture encoding [92] and of electrophysiological theta coherence in ASD contrasting with rightward in TD, during picture recognition [86], contradicts the typical material-specific lateralization of brain activity (visual conceptual representations associated with the right hemisphere, [107]). There are two possible reasons for this atypical leftward lateralization of visual LTM. It may be because of the inclusion of young participants in these two studies [86, 92], leftward lateralization of connectivity during visual processing being particularly evident in younger ASD participants [139]. It could also result from greater white matter integrity of the left inferior longitudinal fasciculus in ASD relative to the right [106].

Second, fMRI and EEG studies point toward a preserved or even enhanced functioning of the posterior structures linked to associative memory, especially the hippocampus. All the fMRI studies on LTM reviewed here that have focused on the hippocampus have identified its similar [82, 89, 90] or even increased [91] activation in ASD compared with TD. This result could be related to the increase in hippocampal volume identified in childhood [140] and adolescence in ASD [141]. In addition, ERP recognition studies have consistently showed diminished or atypically located early mid-central (FN400) potentials in ASD relative to TD, in conjunction with similar [87, 88] or even wider [79] late positive parietal (LPC) potential. This electrophysiological pattern may indicate a greater involvement of recollection over familiarity processes, which, however, contradicts behavioral observations of lower recollective awareness in ASD [38–40], though discrepant results [42]. Instead, as the LPC potential typically indexes associative recognition [26–28] and involves the hippocampus [142], it may rather indicate a greater involvement of associative processes. These ERP results may correspond to an immature development of memory processes, as observed in TD children where episodic recognition is only associated with the LPC potential, stable with age, while the FN400 potential increases to adulthood [28, 143], possibly implicating long-range underconnectivity in ASD that impacts mostly frontal connectivity and preserves cognitive processes subserved by posterior brain areas [122]. Given the binding properties of the hippocampus in LTM, these fMRI and ERP results suggest a greater involvement of relational representations in episodic memory in ASD. This could explain the greater reliance on detailed (verbatim) over global and conceptual (gist-based) memory representations in ASD [144], in contrast to a developmental shift improving gist-based strategies observed in TD individuals across the lifespan [145]. Moreover, a diminished prefrontal activation or prefrontal-hippocampal connectivity co-occurred with this preserved hippocampal activation during relational encoding [82, 91] or retrieval [90] in fMRI studies. Most recent theories in TD suggest complementary learning systems, where the hippocampus encodes moment-to-moment changes in incoming inputs, while the prefrontal cortex integrates over time their similarities into abstract categories; the prefrontal cortex enables a context-guided encoding, via a top-down selection of relevant information by the hippocampus [146, 147]. This process could be reduced in ASD, as evidenced by Cook et al. [92], showing no modulation between medial prefrontal areas and medial temporal lobe activations according to the degree of object–scene congruency at encoding, contrary to the TD control group. Hence, reduced prefrontal–hippocampal connectivity may explain the difficulties of individuals with ASD in identifying similarities between related information [33, 34], and the greater difficulties with more complex stimuli such as sentences or stories [45]. It may also explain the normalization of memory performance in situations providing support for the processing of relational information [33, 36, 37].

Third, two studies reported greater differences between ASD and TD occurring during the recognition than encoding phase. Lynn et al. [83] described a greater reduction in connectivity between the right FFA and the visual cortex during face recognition than during encoding, and Cooper et al. [90] reported diminished frontal activation and connectivity between the hippocampus and fronto-parietal network during visual associative recognition in ASD individuals. During encoding, this last pattern was similar in TD participants. Moreover, while encoding and retrieval in episodic memory typically share common processes [148], reduced post-retrieval verification processes may further hamper recognition, as identified by two ERP studies showing diminished activity or non-specific latency of the related late frontal potential [87, 88].

### Synthesis: specific memory characteristics in ASD linked functional brain asymmetries in the three anatomical planes

**Fig. 2:**
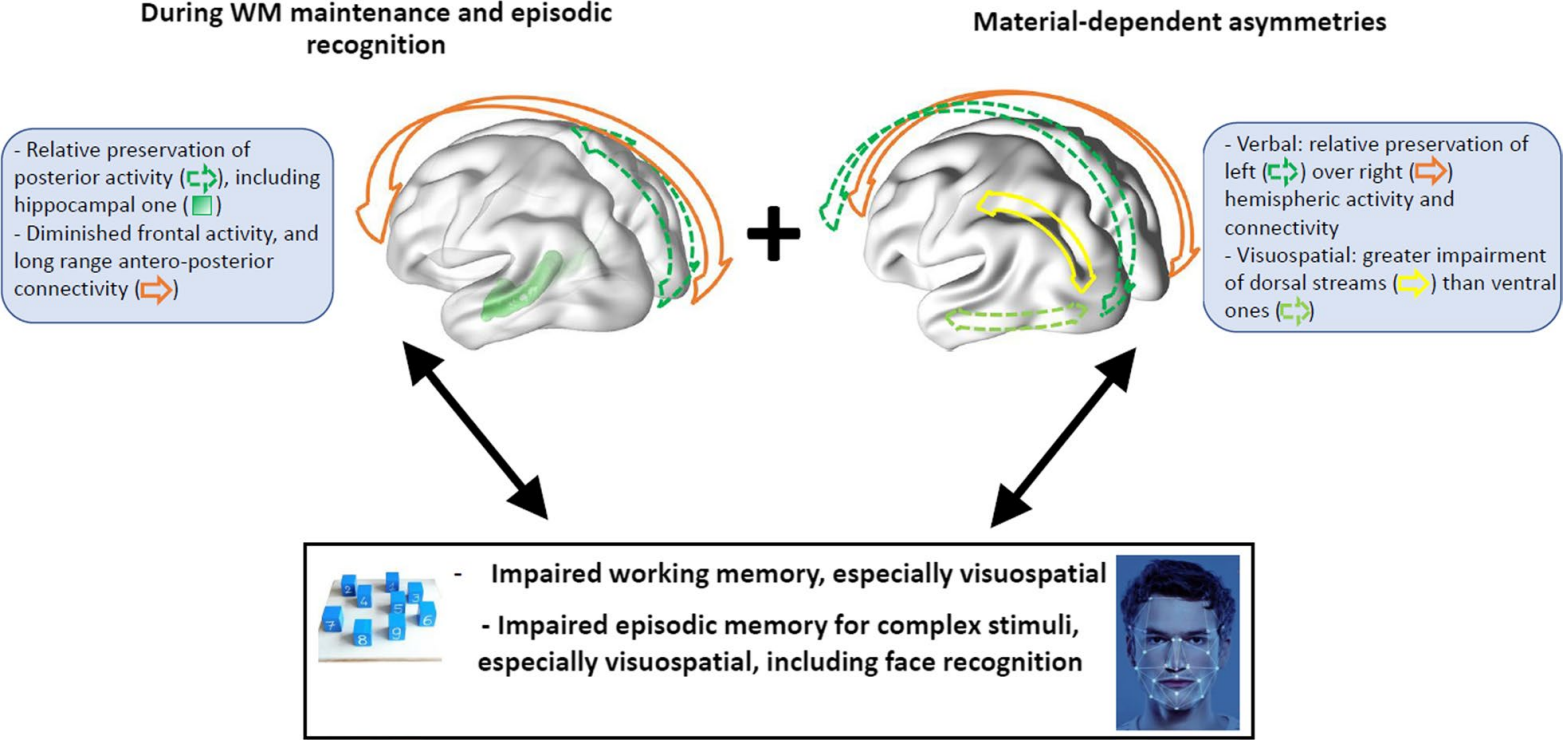
*A summary of brain functional asymmetries during memory processing in ASD*. Brain functional asymmetries during memory processing in ASD are dependent to the material (top right part of the panel): a relative preservation of left over right-hemisphere activity and connectivity for verbal and visuospatial material and a greater impairment of dorsal streams than of ventral ones, for visuospatial material. Apart from these material-dependent effects, functional asymmetries were generally found during working (WM) maintenance and episodic recognition (top left part of the panel): a relative preservation of posterior activity—including a full hippocampal one—and connectivity over frontal activity and antero-posterior long-range connectivity. Interactions between the location of these functional alterations with specialization of brain areas may result in atypical WM (diminished rehearsal process and top-down control, lowered task-set relevance) and episodic memory (reduced context-guided encoding, pre- and post-retrieval processes; high episodic processing of relational information, with lowered general context processing), and underpin the cognitive pattern of memory preservation and difficulties identified in meta-analyses in ASD (lower part of the panel), including impaired—especially visuospatial—WM and episodic memory for complex stimuli, including face recognition

The studies reviewed here highlight functional brain asymmetries between ASD and TD participants during memory tasks in three anatomical planes. These asymmetries consist in greater reliance on the left hemisphere than the right, on posterior structures including the hippocampus rather than anterior brain regions, and on ventral occipito-temporal rather than dorsal occipito-parietal stream. We suggest that interactions between location and severity of these functional alterations with specialization of brain areas are linked to in atypical memory functioning in ASD. Although this functioning relies on the same basic processes as are used in TD individuals, they are employed in a different way. Based on the studies reviewed here, we propose a neuroimaging model of memory functioning in ASD, focusing on these functional asymmetries with assumptions about the autism-specific characteristics of memory processes and representations (Fig. 2).

The first asymmetry may emerge from the relative preservation of left over right intra-hemispheric connectivity, identified from early childhood [149] through to later development in ASD [99–101], and may contribute to the greater verbal over visuospatial memory in ASD highlighted in meta-analyses. This may result in a processing of verbal information in ASD close to that in TD, with a preserved memory representation of these stimuli supported by the semantic memory system, contrasting with reduced configural processing of visuospatial information including faces in ASD, involving more featurally-based representation, less associated with the semantic memory system.

The second asymmetry may result from both the overall long-range underconnectivity in ASD that begins from early development and impacts mostly frontal connectivity [100, 108, 122, 137, 150] and the greater hippocampal volume in ASD compared with TD [140, 141] and may also interfere with memory processes, particularly those involved in WM, as also highlighted by the meta-analyses. It may impair the typical age-dependent reliance of both WM [135, 136] and episodic LTM [151] on frontal areas, attenuating rehearsal and top-down processes that are critical for WM maintenance and the context-guided encoding pre- and post-retrieval processes in LTM, also supporting high-fidelity WM representations and rich relational information in episodic LTM.

The third asymmetry, based on only few results, may be related to dorsal stream dysfunction in ASD also implicating underconnectivity [108, 112] and may further explain the greater difficulties in memory tasks requiring spatial processing of information such as memory for faces.

Intriguingly, within these asymmetries, the brain areas with relatively preserved activity and connectivity in ASD when compared to TD, are those where in humans white matter develops first, during the early phases of life. Structural neuroimaging studies of white matter in TD have shown a leftward asymmetry in fetuses and neonates [152], both posterior-to-anterior and central-to-peripheral direction of maturation during the first few years [153], and earlier maturation in the ventral stream compared with the dorsal stream during childhood [115], which questions the link in ASD between atypical early brain development and later memory functioning.

### Limitations

This review has several limitations. First, although all but one of the reviewed studies reached a high or very high quality on the Kmet score, only half achieved the maximum quality for the appropriate sample size. Hence, a low number of participants in reviewed studies may limit the robustness of their brain imaging results. Second, diagnostic criteria and psychometric data for participants with ASD were heterogeneous across studies, which may have induced variability in neuroimaging results. Indeed, the IQ of individuals with ASD is a significant factor influencing differences in memory performance between groups with ASD and TD [5], and it also shows a substantial variability in their development into adulthood [154]. The age ranges of participants were sometimes wide, both within and between studies. In addition, no neuroimaging study of memory has been conducted in individuals with ASD and language impairment (ASD-LI), although behavioral studies have evidenced a specific functioning of their declarative memory [155]. Third, while the methodology of the WM studies was relatively homogeneous, based on N-back tasks, the methodology of LTM studies was heterogeneous, which made it more difficult to identify a clear pattern of results. In both cases, there was little use of verbal memory tests, which is a weakness of neuroimaging memory studies in ASD, especially given that academic learning relies in substantial part on this type of memory. Moreover, the studies reviewed here mainly used recognition tasks, on which individuals with ASD perform similarly to those with TD [5], while free recall tasks, for which ASD individuals experience more difficulty, have barely been explored in neuroimaging. The combined variability of reviewed studies regarding the age of participants, the memory tasks used, the stage of the memory process investigated, as well as the nature of the neural signal acquired, made it not possible to produce a quantitative (meta-analytic) confirmation of the synthesis. Fourth, the proposed neuroimaging model is based on functional differences between ASD and TD, including brain activity and connectivity, that presumably are influenced by structural differences in gray and white matter. However, none of reviewed studies included structural measures, making it unclear to what extent structural abnormalities account for the functional differences between ASD and TD groups. Finally, the interpretations of the differences between ASD and TD concerning memory processes and representations are based on the presumption of identical brain specialization, which remains difficult to confirm.

### Conclusions and future directions

On the basis of the studies reviewed here, we proposed a neuroimaging model of memory in ASD based on functional asymmetries (i.e., of activity and connectivity) in three anatomical planes. We related these asymmetries to anatomical and structural connectivity alterations commonly identified in ASD. This neuroimaging model helps explain the pattern of behavioral results depending on material or type of memory in several meta-analyses. Although this model has the potential to unify widely disparate results, it also faces several limitations, including lack of quantitative (meta-analytic) confirmation. Future research could confirm or improve this model, by combining measures of structural and functional connectivity in same memory paradigms and by varying type of memory, type of material, and level of complexity of information in same participants.

## Data Availability

Data sharing is not applicable to this article as no datasets were generated during the current study.

## Appendix

Quality assessment of included WM and episodic LTM studies (Kmet, Lee, & Cook, 2004, checklist). Scores: 2 = Yes, 1 = Partial, 0 = No, N/A = not applicable.

**Table:**

Kmet, Lee, & Cook (2004) checklist for assessing the quality of quantitative studies	Audrain et al. [67]	Barendse et al. [68]	Braden et al. [64]	Chan et al. [86]	Chantiluke et al. [59]	Churches et al. [81]	Cook et al. [92]	Cooper et al. [90]	Desaunay et al., [79]	Gaigg, et al. [89]	Greimel et al., [82]	Gunji et al. [80]
1. Question/objective sufficiently described?	2	2	2	2	2	2	2	2	2	2	2	2
2. Study design evident and appropriate?	2	2	2	2	2	2	2	2	2	2	2	2
3. Method of subject/comparison group selection or source of information/input variables described and appropriate?	1	2	2	2	2	2	2	2	2	2	2	2
4. Subject (and comparison group, if applicable) characteristics sufficiently described?	2	2	2	2	2	1	2	2	2	2	2	1
5. If interventional and random allocation was possible, was it described?	N/A	N/A	N/A	N/A	2	N/A	N/A	N/A	N/A	N/A	N/A	N/A
6. If interventional and blinding of investigators was possible, was it reported?	N/A	N/A	N/A	N/A	1	N/A	N/A	N/A	N/A	N/A	N/A	N/A
7. If interventional and blinding of subjects was possible, was it reported?	N/A	N/A	N/A	N/A	1	N/A	N/A	N/A	N/A	N/A	N/A	N/A
8. Outcome and (if applicable) exposure measure(s) well defined and robust to measurement/misclassification bias? Means of assessment reported?	2	2	2	2	2	2	2	2	2	2	2	2
9. Sample size appropriate?	2	1	1	2	1	1	1	2	2	1	1	0
10. Analytic methods described/justified and appropriate?	2	2	2	2	2	2	2	2	2	2	2	2
11. Some estimate of variance is reported for the main results?	2	2	2	2	2	2	2	2	2	2	2	2
12. Controlled for confounding?	2	2	2	2	2	2	2	2	2	2	2	2
13. Results reported in sufficient detail?	2	2	2	2	2	2	2	2	2	2	2	2
14. Conclusions supported by the results?	2	2	2	2	2	2	2	2	2	2	2	2
Total score	21	21	21	22	25	20	21	22	22	21	21	19
Percentage	95%	95%	95%	100%	89%	91%	95%	100%	100%	95%	95%	86%

**Table:**

Kmet, Lee, & Cook (2004) checklist for assessing the quality of quantitative studies	Hawco et al. [69]	Herrington et al. [55]	Hogeveen et al. [91]	Kleinhans et al. [57]	Koshino et al. [51]	Koshino et al. [56]	Larrain-Valenzuela et al. [66]	Luna et al. [60]	Lynn et al. [83]	Massand et al. [87]	Massand & Bowler [88]
1. Question/objective sufficiently described?	2	2	2	2	2	2	2	2	2	2	2
2. Study design evident and appropriate?	2	2	2	2	2	2	2	2	2	2	2
3. Method of subject/comparison group selection or source of information/input variables described and appropriate?	1	2	2	2	1	1	2	1	1	1	2
4. Subject (and comparison group, if applicable) characteristics sufficiently described?	2	2	2	2	2	2	2	1	2	1	2
5. If interventional and random allocation was possible, was it described?	N/A	N/A	N/A	N/A	N/A	N/A	N/A	N/A	N/A	N/A	N/A
6. If interventional and blinding of investigators was possible, was it reported?	N/A	N/A	N/A	N/A	N/A	N/A	N/A	N/A	N/A	N/A	N/A
7. If interventional and blinding of subjects was possible, was it reported?	N/A	N/A	N/A	N/A	N/A	N/A	N/A	N/A	N/A	N/A	N/A
8. Outcome and (if applicable) exposure measure(s) well defined and robust to measurement/misclassification bias? Means of assessment reported?	2	2	2	2	2	2	2	2	2	2	2
9. Sample size appropriate?	2	1	2	2	1	1	2	1	2	1	1
10. Analytic methods described/justified and appropriate?	2	2	2	2	2	2	2	2	2	2	2
11. Some estimate of variance is reported for the main results?	2	2	2	1	2	2	2	2	2	2	2
12. Controlled for confounding?	2	2	2	2	2	2	2	2	2	2	2
13. Results reported in sufficient detail?	2	2	2	2	2	2	2	2	2	2	2
14. Conclusions supported by the results?	2	2	2	2	2	2	2	2	2	2	1
Total score	21	21	22	21	20	20	22	19	21	19	20
Percentage	95%	95%	100%	95%	91%	91%	100%	86%	95%	86%	91%

**Table:**

Kmet, Lee, & Cook (2004) checklist for assessing the quality of quantitative studies	Neumann et al. [78]	Noonan, Haist, & Müller [85]	O’Hearn et al. [84]	Rahko et al. [63]	Silk et al. [62]	Urbain, Pang, & Taylor [54]	Urbain et al. [58]	Vogan et al. [52]	Vogan et al. [61]	Vogan et al. [53]	Yuk et al. [65]
1. Question/objective sufficiently described?	2	2	2	2	2	2	2	2	2	2	2
2. Study design evident and appropriate?	2	2	2	2	2	2	2	2	2	2	2
3. Method of subject/comparison group selection or source of information/input variables described and appropriate?	2	2	1	2	2	1	1	1	1	1	1
4. Subject (and comparison group, if applicable) characteristics sufficiently described?	2	2	2	1	2	2	2	2	2	2	2
5. If interventional and random allocation was possible, was it described?	N/A	N/A	N/A	N/A	N/A	N/A	N/A	N/A	N/A	N/A	N/A
6. If interventional and blinding of investigators was possible, was it reported?	N/A	N/A	N/A	N/A	N/A	N/A	N/A	N/A	N/A	N/A	N/A
7. If interventional and blinding of subjects was possible, was it reported?	N/A	N/A	N/A	N/A	N/A	N/A	N/A	N/A	N/A	N/A	N/A
8. Outcome and (if applicable) exposure measure(s) well defined and robust to measurement/misclassification bias? Means of assessment reported?	2	2	2	2	2	2	2	2	2	2	2
9. Sample size appropriate?	0	1	2	2	0	2	2	2	2	2	2
10. Analytic methods described/justified and appropriate?	2	2	2	2	2	2	2	2	2	2	2
11. Some estimate of variance is reported for the main results?	2	2	2	1	1	2	2	2	2	2	2
12. Controlled for confounding?	2	2	2	2	1	2	2	2	2	2	2
13. Results reported in sufficient detail?	2	2	2	2	1	2	2	2	2	2	2
14. Conclusions supported by the results?	2	2	2	2	2	2	2	2	2	2	2
Total score	20	21	21	20	17	21	21	21	21	21	21
Percentage	91%	95%	95%	91%	77%	95%	95%	95%	95%	95%	95%

## Abbreviations

ASD: Autism spectrum disorder
DMN: Default-mode network
EEG/MEG: Electro-/magnetoencephalography
ERP: Event-related potential
FFA: Fusiform face areas
fMRI: Functional magnetic resonance imaging
FN400 potential: Frontal, negative, around 400 ms
LTM: Long-term memory
LPC potential: Late positive (or parietal) component
TD: Typical development
WM: Working memory
